# The Efficacy of Different Voice Treatments for Vocal Fold Polyps: A Systematic Review and Meta-Analysis

**DOI:** 10.3390/jcm12103451

**Published:** 2023-05-13

**Authors:** Ben Barsties v. Latoszek, Christopher R. Watts, Svetlana Hetjens, Katrin Neumann

**Affiliations:** 1 Speech-Language Pathology, SRH University of Applied Health Sciences, 40210 Düsseldorf, Germany; 2 Harris College of Nursing & Health Sciences, Texas Christian University, Fort Worth, TX 76109, USA; 3 Department for Medical Statistics and Biomathematics, Medical Faculty Mannheim, University of Heidelberg, 68165 Mannheim, Germany; 4 Department of Phoniatrics and Pediatric Audiology, University Hospital Münster, University of Münster, 48149 Münster, Germany; katrin.neumann@uni-muenster.de

**Keywords:** vocal fold polyp, voice treatment, voice therapy, surgery, auditory–perceptual judgment, jitter, shimmer, maximum phonation time, voice handicap index

## Abstract

Background: Vocal fold polyps (VFP) are a common cause of voice disorders and laryngeal discomfort. They are usually treated by behavioral voice therapy (VT) or phonosurgery, or a combination (CT) of both. However, the superiority of either of these treatments has not been clearly established. Methods: Three databases were searched from inception to October 2022 and a manual search was performed. All clinical trials of VFP treatment were included that reported at least auditory–perceptual judgment, aerodynamics, acoustics, and the patient-perceived handicap. Results: We identified 31 eligible studies (VT: n = 47–194; phonosurgery: n = 404–1039; CT: n = 237–350). All treatment approaches were highly effective, with large effect sizes (*d* > 0.8) and significant improvements in almost all voice parameters (*p*-values < 0.05). Phonosurgery reduced roughness and NHR, and the emotional and functional subscales of the VHI-30 were the most compared to behavioral voice therapy and combined treatment (*p*-values < 0.001). Combined treatment improved hoarseness, jitter, shimmer, MPT, and the physical subscale of the VHI-30 more than phonosurgery and behavioral voice therapy (*p*-values < 0.001). Conclusions: All three treatment approaches were effective in eliminating vocal fold polyps or their negative sequelae, with phonosurgery and combined treatment providing the greatest improvement. These results may inform future treatment decisions for patients with vocal fold polyps.

## 1. Introduction

Vocal fold polyps (VFP) are functional voice disorders associated with benign tissue changes to the vocal folds. They are commonly unilateral. Their shape can be classified as sessile or peduncular; their morphological characteristics can be classified as gelatinous or translucent, fibrous or organized, and angiomatous or hemorrhagic [1]. Their size can vary from small to medium to large (<¼ of the vocal fold length, ¼–⅓, >⅓) [2]. In some regions, VFP is among the five most common laryngeal diseases, with prevalence figures of 0.4–9% [3,4,5]. VFP are caused primarily by a coincidence of non-physiologic voice use, i.e., “phonotrauma” (inflammatory response of the vocal fold mucosa to biomechanical stress and deformations during high-effort vibration) often associated with yelling or awkward singing, and other etiological factors such as upper respiratory tract infections, allergies, gastroesophageal reflux, and smoking [1,6]. Patients with VFP can experience significant impairment in phonation and communication, with negative social implications. Usually, voice complaints due to VFP involve dysphonia, increased vocal effort, and decreased vocal stamina. Dysphonia associated with VFP results from complex changes in the vibrational patterns of the vocal folds through alterations in their layered structure and stiffness of tissue. VFP require multidimensional voice assessments (vocal fold imaging, auditory–perceptual judgment, acoustic and aerodynamic measurements, and patients’ self-evaluation) [7]. Their results may be influenced by the size, form, mass, and base length of the polyp and the resulting changing area and shape of the glottal gap during phonation [8]. In the treatment of VFPs, phonosurgery is often the first choice [9,10], but behavioral voice therapy (VT) is also recommended as an effective treatment modality, either as a stand-alone treatment or combined with phonosurgery [11]. The efficacy of VT can be explained, in part, by a causal role of hypertension of the laryngeal musculature and, in particular, supraglottic structures in the development of the polyps. Although clinical guidelines recommend treating VFP conservatively first and resecting them secondarily only if results are unsatisfactory, the efficacy of phonosurgery as a primary treatment option for VFP has been confirmed by observational studies [12]. The recurrence rate of VFP after surgery has been reported as low (11%) and not influenced by gender but by age (younger adults have a significantly higher relapse rate than middle-aged or older adults) [13]. The choice of treatment option for VFP is important because either the risks and costs of surgery can be avoided if VT is the treatment of first choice or phonosurgery can lead to the faster recovery of vocal function. Therefore, the aim of this meta-analysis was to compare the efficacy of phonosurgery, VT, and a combination of both (CT) in the treatment of VFP based on multidimensional voice assessments pre- and post-treatment.

## 2. Materials and Methods

### 2.1. Data Sources and Searches

We followed the Preferred Reporting Items for Systematic Reviews and Meta-Analyses (PRISMA) guidelines [14] and systematically searched four databases (MEDLINE, CENTRAL, CINAHL, and KoreaScience) from inception to 26 October 2022 (Table S1, Supplementary Materials). Potentially eligible publications, including those published in different languages from the above databases, were identified by title and abstract. In addition, a manual search of congress proceedings, grey literature, and bibliographies was performed.

### 2.2. Study Selection

All available intervention trials with a pre-post design for VFP using phonosurgery alone, VT alone, or a combination with initial phonosurgery followed by VT were included in the search. According to a preliminary search, the combination of initial VT followed by phonosurgery (investigated minimally in multiple case studies) was low (n = 1); thus, we excluded it from our meta-analysis. The clinical parameters of this meta-analysis included the most commonly used quantitative measures of an internationally agreed battery of voice examinations [7]: auditory–perceptual voice assessment (hoarseness, breathiness, roughness) by at least one examiner using a Likert scale ranging from 0 (no impairment) to 3 (maximum impairment), acoustic (jitter, shimmer, noise-to-harmonics ratio NHR) and aerodynamic (maximum phonation time, MPT) measures, and a standardized questionnaire for the self-assessment of voice handicap. To avoid specification and reliability differences due to the application of different acoustic software packages, only studies that performed acoustic measurements with the Multi-Dimensional Voice Program (Kay Elemetrics Corporation, Lincoln Park, NJ, USA) were included. To make self-assessments of vocal handicap comparable, we included only studies that assessed it with the most widely used international standardized questionnaire, the 30-item Voice Handicap Index (VHI—30) [15], which outputs three subscales with statements on physical (P), functional (F), and emotional (E) domains and a total score (T). Studies eligible for the meta-analysis had to involve at least one of the aforementioned measures.

### 2.3. Risk of Bias Assessments

The risk of bias assessment of the included studies was determined using the RoB 2 tool [16] for randomized studies (overall risk ranging from low to some concerns to high) and ROBINS-I tool [17] for non-randomized studies (overall risk ranging from low to moderate to serious to critical to no information).

### 2.4. Statistics

Statistical analyses were completed using MedCalc software (version 19.6) and SAS software, release 9.4 (Cary, NC, USA). At first, the difference between the mean values ${\bar{x}}_{post}-{\bar{x}}_{pre}$ and standard error (SE) was calculated as $\mathrm{SE}=\frac{(S_{1}+S_{2})/2}{\sqrt{n}}$. Thereafter, the meta-analysis with weighting based on the random effects model was performed using MedCalc software (version 19.6) by treatment and dysphonia measures. The mean pre-post treatment differences of voice measures with a 95% confidence interval (95% CI) per study and pooled analyses are shown in forest plots. The heterogeneity of studies was calculated using the I² index (0–25% insignificant, >25–50% low, >50–75% moderate, >75% high heterogeneity) [18]. The random effects model was used to analyze the pooled data, accounting for heterogeneity between studies. Studies were weighted according to DerSimonian and Laird [19]. Potential publication bias was analyzed using Egger’s test [20].

To potentially reduce heterogeneity in treatment outcomes and refine them, four subgroup analyses were performed for phonosurgery and CT (fewer studies were available for VT). Subgroup 1 was formed according to the time interval between pre- and postoperative measurements for polyp resection, divided into three follow-up periods: ≤1 month; 1–2 months; ≥3 months. Subgroup 2 was established by the type of three phonosurgical techniques used: cold knife; laser; a combination of cold knife and laser. Subgroup 3 was based on one of two types of surgical techniques combined with VT: cold knife; laser. Subgroup 4 was defined according to one of two durations of VT after phonosurgery: 1–2 weeks; >3 weeks.

A network meta-analysis between treatment approaches was then conducted using SAS software. This involved comparing the pooled mean pre-to-post-treatment differences among the three interventions, along with their confidence intervals, using the Satterthwaite *t*-test. In case of a significant result, the ranking of treatment was based on the highest mean pre- to post-treatment difference in results. Cohen’s *d* was calculated as the effect size of treatment approaches and voice measures, whereby convention 0.2–0.5 is considered a small effect, 0.5–0.7 is considered a medium effect, and >0.8 is considered a large effect [21].

## 3. Results

**Figure 1:**
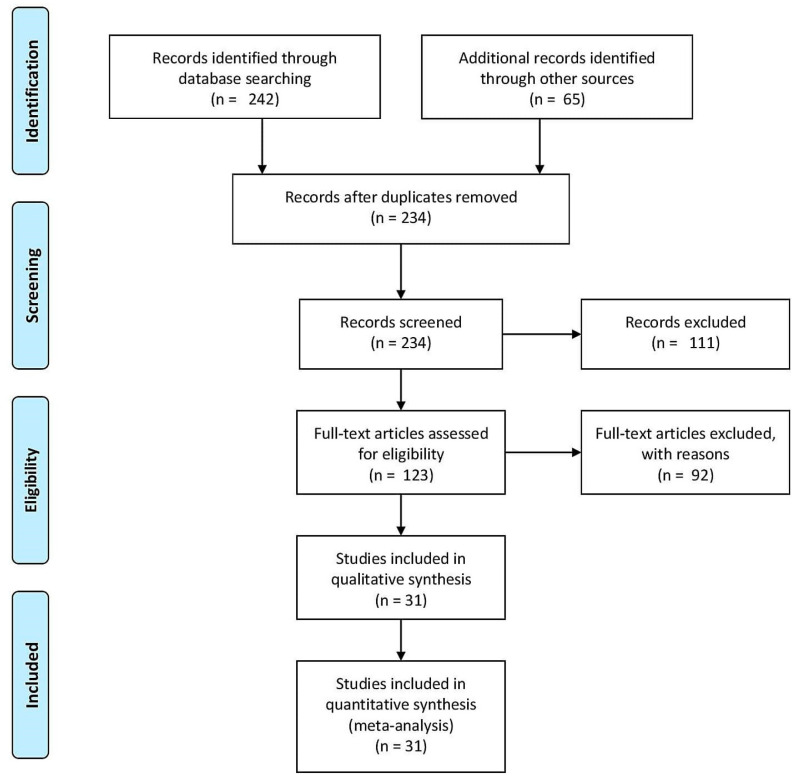
PRISMA flow diagram.

We identified 234 non-duplicates from our searches (Figure 1). Of these, 31 studies were eligible for inclusion in this review (Table 1) [22,23,24,25,26,27,28,29,30,31,32,33,34,35,36,37,38,39,40,41,42,43,44,45,46,47,48,49,50,51,52]. The results of the risk of bias analysis are shown in Table S2, Supplementary Materials. For the randomized trials, the risk of bias was low for one study [42] and some concerns existed for two studies [35,39]. For the observational studies, the overall risk of bias was low for one study [40], moderate for nine studies [28,29,33,37,41,43,50,51,52], and serious for eighteen studies [22,23,24,25,26,27,28,30,31,32,34,36,44,45,46,47,48,49]. The results of the meta-analysis with heterogeneity statistics and publication bias analyses are shown in Table S3, Supplementary Materials.

### 3.1. Auditory–Perceptual Judgment

**Figure 2:**
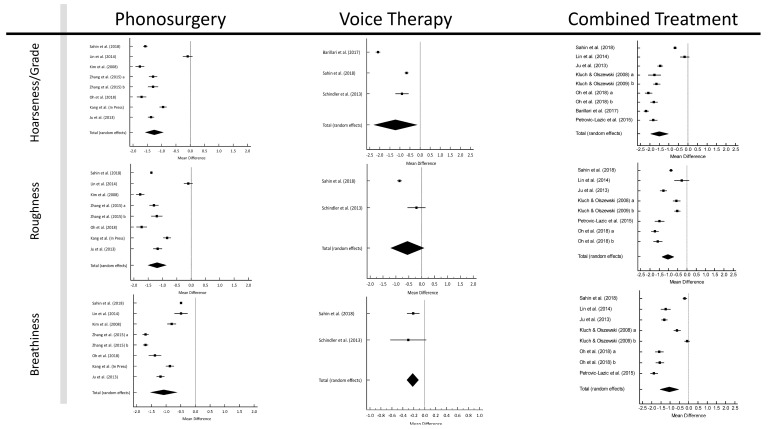
Forest plots of perceived voice quality levels [23,24,26,30,31,35,38,39,42,45,46,52].

This meta-analysis used the G (grade [of dysphonia]), R (roughness), and B (breathiness) parameters of the international GRBAS scale [7]. Its forest plots are shown in Figure 2. The pooled pre- to post-treatment gains of G were −1.256 (95% CI: −1.569–−0.944; *p* < 0.001) for phonosurgery, −1.223 (95% CI: −2.293–−0.152; *p* = 0.025) for VT, and −1.504 (95% CI: −1.972–−1.037; *p* < 0.001) for CT, indicating that all interventions reduced hoarseness. All Cohen’s *d* values were above 0.8 (Table S4, Supplementary Materials).

There was no significant publication bias for these analyses (*p* > 0.05). Heterogeneity was high (>75%) and persisted in the subgroup analyses (follow-up period and type of phonosurgery) (Table S6, Supplementary Materials). The longer the duration of the follow-up period after phonosurgery, the lower the heterogeneity, but it remained >75%. The mean G reduced the most with CT after 1–2 weeks of follow-up, at −1.638 (95% CI: −1.810–−1.466; *p* < 0.001), and even at a moderate heterogeneity of 64.96%. In the network meta-analysis, G improved more for CT than for phonosurgery alone (*p* < 0.001) or VT (*p* < 0.001), with no significant differences across studies (*p* = 0.518; Table S5, Supplementary Materials). The pooled roughness was −1.189 (95% CI: −1.505–−0.944; *p* < 0.001) for phonosurgery, −0.552 (95% CI: −1.198–−0.093; *p* = 0.093) for VT, and −1.041 (95% CI: −1.361–−0.722; *p* < 0.001) for CT. All Cohen’s *d* values were above 0.8 (Table S4, Supplementary Materials). A significant publication bias was evident for VT (*p* < 0.001). Heterogeneity was high (>75%) but steadily decreased with increasing duration of follow-up, reaching a moderate value of 54.11% ≥ 3 months after surgery (Table S6, Supplementary Materials). A network meta-analysis for all treatment modalities showed a significant reduction in roughness, with phonosurgery being the most effective (*p* < 0.001) (Table S5, Supplementary Materials). The pooled breathiness value was −1.080 (95% CI: −1.529–−0.630; *p* < 0.001) for phonosurgery, −0.220 (95% CI: −0.327–−0.113; *p* < 0.001) for VT, and −1.055 (95% CI: −1.557–−0.553; *p* < 0.001) for CT. All Cohen’s *d* values were above 0.8 (Table S4, Supplementary Materials). A significant publication bias was evident for VT (*p* < 0.001) and CT (*p* = 0.018). Heterogeneity was 0% for VT but was high (>75%) for phonosurgery and CT and remained high in subgroup analyses (follow-up period and type of phonosurgery; Table S6, Supplementary Materials).

The network meta-analysis showed significant outcome differences between phonosurgery and VT (*p* < 0.001) and VT and CT (*p* < 0.001) but not between phonosurgery and CT (*p* = 0.198), both of which were most effective (Table S5, Supplementary Materials).

### 3.2. Acoustics

**Figure 3:**
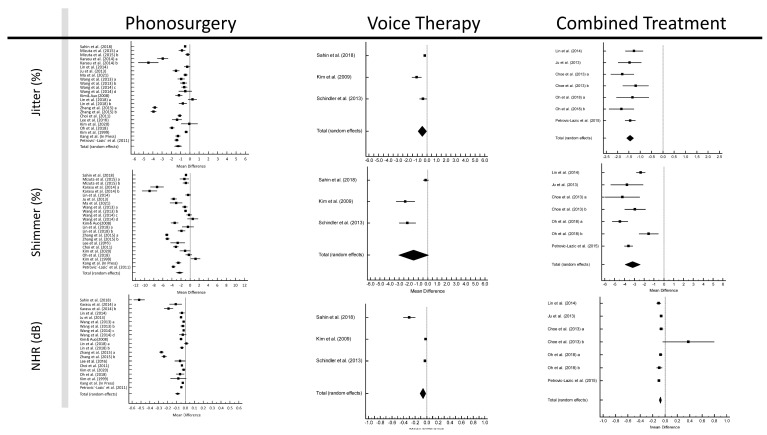
Forests plot of acoustic measures [22,23,27,28,29,30,31,32,33,34,35,36,37,38,39,40,44,45,46,49,50,52].

Forest plots for this meta-analysis are depicted in Figure 3. The pooled pre- to post-treatment jitter differences were −1.266% (95% CI: −1.663–−0.869%; *p* < 0.001) for phonosurgery, −0.494% (95% CI: −0.932–−0.057%; *p* < 0.001) for VT, and −1.457% (95% CI: −1.615–−1.299%; *p* < 0.001) for CT. The pooled pre–post shimmer differences were −2.300% (95% CI: −3.061–−1.539%; *p* < 0.001) for phonosurgery, −1.487% (95% CI: −3.065–0.092%; *p* < 0.001) for VT, and −3.181% (95% CI: −3.950–−2.413%; *p* < 0.001) for CT. The pooled pre-post NRH differences were −0.087 dB (95% CI: −0.113–−0.061 dB; *p* < 0.001) for phonosurgery, −0.068 dB (95% CI: −0.118–−0.017 dB; *p* < 0.001) for VT, and −0.077 dB (95% CI: −0.096–−0.059 dB; *p* < 0.001) for CT. All Cohen’s *d* values were above 0.8 (Table S4, Supplementary Materials).

For shimmer in the VT analysis and jitter in the phonosurgery analysis, there were significant publication biases (*p* = 0.026 and *p* = 0.045, respectively). Heterogeneity was high for phonosurgery and VT but low for CT (Table S3, Supplementary Materials), with comparable results in the subgroup analyses (Table S7, Supplementary Materials). For jitter, the heterogeneity for CT even reached 0%. For all three parameters, the mean gain after phonosurgery gains was greatest for a follow-up period of ≥3 months: jitter: −2.166% (95% CI: −3.925–−0.408%; *p* = 0.016), NHR: −0.339 dB (95% CI: −0.452–−0.225 dB; *p* < 0.001), shimmer: −2.646% (95% CI: −5.039–−0.252%; *p* = 0.030).

In the network meta-analysis, the pooled pre–post improvements of all three acoustic parameters were significant for all treatment modalities, with jitter and shimmer showing the strongest improvements with CT and NHR showed the strongest improvements with phonosurgery (all *p* < 0.001; Table S5, Supplementary Materials).

### 3.3. Maximum Phonation Time

**Figure 4:**
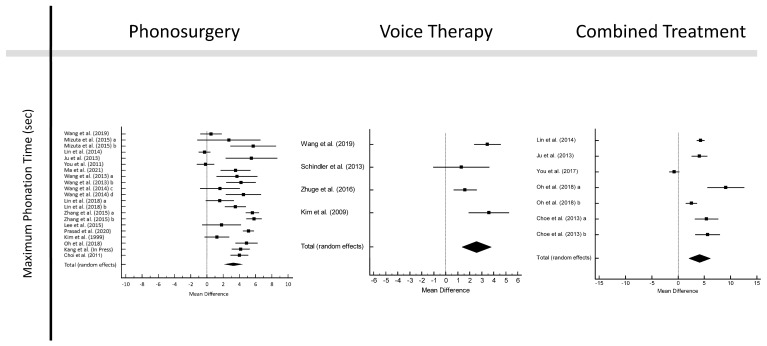
Forest plots of maximum phonation time [22,27,28,30,31,32,33,35,36,37,39,40,41,43,44,46,47,48,50,52].

The pooled pre- to post-treatment MPT elongations were 3.265 s (95% CI: 2.203–4.328 s; *p* < 0.001) for phonosurgery, 2.561 s (95% CI: 1.355–3.766 s; *p* < 0.001) for VT, and 4.065 s (95% CI: 2.045–6.084 s; *p* < 0.001) for CT (forest plots in Figure 4). All Cohen’s *d* values were above 0.8 (Table S4, Supplementary Materials).

There was no significant publication bias (*p* > 0.05). Heterogeneity was lowest for VT with I^2^ = 65.80%. In the subgroup analyses (Table S8, Supplementary Materials), the mean pre–post gain was highest for CT if administered for longer than 3 weeks (4.521 s; 95% CI: 1.436–7.606 s; *p* = 0.004), but heterogeneity was high (95.55%). High pooled MPT gains of 4.468 s (95% CI: 3.632–5.303 s; *p* < 0.001) with insignificant heterogeneity of 7.26% was achieved for CT with phonosurgical laser technology.

In the network meta-analysis, pooled pre–post MPT prolongation was significant for all treatment modalities and highest for CT (*p* < 0.001; Table S5, Supplementary Materials).

### 3.4. Voice Handicap Index—30

**Figure 5:**
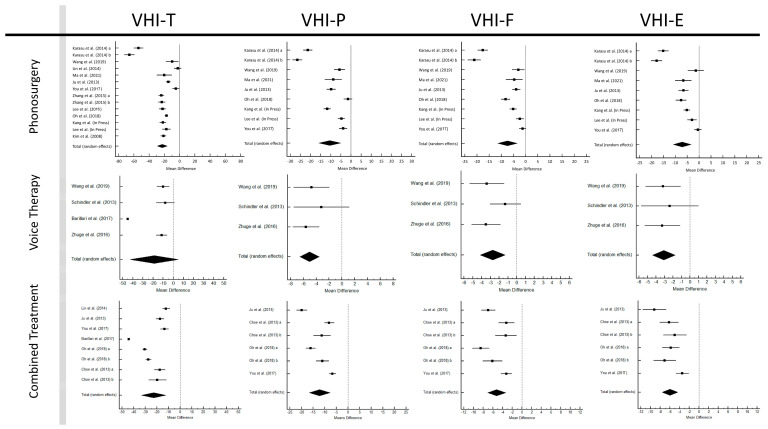
Forest plots of Voice Handicap Index parameters [25,30,31,32,34,35,39,40,41,42,43,46,47,50,51,52].

Forest plots are shown in Figure 5. The pooled pre–post improvements of the E (emotional) subscale values were −7.072 (95% CI: −10.786–−3.357; *p* < 0.001) for phonosurgery, −3.093 (95% CI: −4.440–−1.747; *p* < 0.001) for VT, and −6.242 (95% CI: −11.913–−0.571; *p* = 0.031) for CT. The pooled pre–post gains of the F (functional) subscale values were −7.437 (95% CI: −11.389–−3.485; *p* < 0.001) for phonosurgery, −2.731 (95% CI: −4.162–−1.300; *p* < 0.001) for VT, and −5.239 (95% CI: −7.124–−3.354; *p* < 0.001) for CT. The pooled pre-to-post treatment P (physical) subscale enhancements were −10.463 (95% CI: −15.829–−5.096; *p* < 0.001) for phonosurgery, −5.022 (95% CI: −6.569–−3.476; *p* < 0.001) for VT, and −12.200 (95% CI: −16.668–−7.731; *p* < 0.001) for CT. The pooled pre–post T (total) score gains were −22.753 (95% CI: −29.266–−16.240; *p* <0.001) for phonosurgery, −18.886 (95% CI: −42.996—5.224; *p* = 0.125) for VT, and −22.896 (95% CI: −33.529–−12.264; *p* < 0.001) for CT. All Cohen’s *d* values were above 0.8 (Table S4, Supplementary Materials).

Significant publication biases were evident for the E subscale for CT (*p* < 0.001), P subscale for VT (*p* = 0.027), and T score for VT (*p* = 0.017) and CT (*p* < 0.001). The E, F, and P subscales scores showed low to moderate heterogeneity for VT (0—48.9%). High T score heterogeneity was present for all treatment modalities, with predominantly high heterogeneity in all subgroup analyses of the VHI parameters for CT and phonosurgery (Table S9, Supplementary Materials). For all four parameters, the mean pre- to post-phonosurgery gains were greatest with a follow-up of 1–2 months: E subscale: −11.106 (95% CI: −17.278–−4.935; *p* < 0.001), F subscale: −11.875 (95% CI: −19.680–−4.070; *p* = 0.003), P subscale: −17.370 (95% CI: −24.860–−9.879; *p* < 0.001), T score: −35.674 (95% CI: −52.365–−18.982; *p* < 0.001). Comparing phonosurgery with CT, the network meta-analysis showed significant improvements (*p* < 0.001, Table S5, Supplementary Materials) for all four VHI parameters, except for the T scores (*p* = 0.674).

## 4. Discussion

This meta-analysis showed that phonosurgery, VT, and a sequential combination of both resulted in significant voice improvements in the treatment of vocal fold polyps, with either phonosurgery alone or phonosurgery followed by VT being the most effective treatment options, with not much difference. Subgroup analyses did not significantly reduce heterogeneity.

To optimize treatment pathways, it would be desirable to include morphologic features of VFP in treatment decisions. We therefore performed an extra subgroup analysis with regard to the morphological characteristics of polyps using the twelve studies available for this purpose. The results are sobering and suggest that further research is needed on this clinical issue. There were six studies that differentiated somewhat in morphologic characteristics but with no common intersection on these characteristics, let alone even two studies per treatment format. These six studies describe the following criteria: bilateral polyps only; unilateral, but no giant polyps; all features and sizes, but polyps must be positioned at the free edge; all types and features of gelatinous polyps; variable sizes; and additional different features in two studies. Six studies further defined the size of the polyp; these studies considered only small to medium-sized polyps. Three studies analyzed voice therapy and phonosurgery and one study analyzed their combination. Thus, no comparative analysis could be performed across all three treatment modalities. By testing VT and phonosurgery in the small to medium polyp sizes, another problem occurred: these six studies did not analyze all 11 of our chosen measures. The only intersection between all studies was maximum phonation time. However, as shown in Table S10, Supplementary Materials, MPT improved on average by the same amount with both methods, namely, by 2.90 s each, with comparable SD. Thus, although the sample was large, with n = 100 patients for each of the two groups examined, there was no clinical difference between phonosurgery and VT in MPT.

Included publications revealed some serious concerns about the risk of bias for many (20 out of 31) studies and high heterogeneity, including in the subgroups. There was imprecision only in the VT group for all voice parameters and publication bias in some cases, but no indirectness.

Our meta-analysis evaluated only the combination of phonosurgery followed by VT, as our search found only one study [45] reporting initial VT followed by phonosurgery if VT did not result in sufficient voice improvement. Nonetheless, the latter treatment modality is recommended in current clinical guidelines for hoarseness [12]. According to our results, VT alone may also be an effective treatment option for VFP, but showed less improvement in voice measures than the other methods. Therefore, the efficiency of initial VT may also be questioned. Moreover, the efficiency of initial or sole VT has been less well studied than that of the other treatment modalities, and the meta-analysis could only rely on a smaller number of participants and less variability in VT. Additionally, it is still unclear which voice exercises in VFP particularly facilitate behavioral changes in voice use or improved voice function. Two recent network meta-analyses identified four VT programs as effective: stretch-and-flow phonation, resonant voice, vocal function exercises, and an eclectic VT program [53,54]. Strong, direct VT concepts for VFP might include VT expulsion [42], Seong-Tae Kim’s multiple VT technique [27], vocal function exercises [41,42,43,45,46], and resonant voice [30,31,32,41,43,45,46]. Furthermore, polyp characteristics such as small size influence the success of VT and should be considered [2,55]. Further research is needed to clarify the effectiveness of the named VT methods, depending on VFP characteristics. Moreover, vocal hygiene, including environment change (e.g., humidifier in dry air, mask in dusty air, amplification in a noisy environment), behavior change (e.g., avoiding lifting/pushing heavy things, loud coughing, throat clearing, excessive alcohol and caffeine consumption, smoking, late meals, fatty and spicy foods), and vocal habits change (e.g., avoiding shouting, speaking with anger, loud whispering), is imperative after VFP phonosurgery [26,30,31,32,35,41,42,43,45,46,47].

In clinical practice, polyp-like masses of the vocal folds are occasionally not given names when diagnosed by stroboscopy or laryngoscopy because of uncertainty about their histologic nature or dignity (e.g., thin-walled cyst, atypical laryngeal carcinoma); these patients are then referred to a laryngeal surgeon with diagnoses such as “unclear lesion of the vocal fold.” In these cases, phonosurgical ablation is the method of choice. Thus, in the cases of VT alone, our meta-analysis carries some uncertainty as to whether polyps were really involved. However, that this possibility may have had only a small impact is shown by the large effect of VT.

Most studies included in our meta-analysis used the VHI-30 as a self-assessment measure, and only seven studies applied the VHI-10, which is also often utilized in routine clinical practice [56]. Of these, five studies were already included in this meta-analysis based on other measures of acoustics, auditory–perceptual judgment, or aerodynamics. For inclusion in a meta-analysis, the VHI-10 would have had to be used in more than one study for all three treatment modalities, a constellation that was not found in our search. Nevertheless, it is useful to consider a VHI that is standardized in terms of its item numbers in clinical and research evaluations of voice treatments, and we encourage readers to invest more in standardized multidimensional voice assessments to achieve better comparability of treatment outcomes.

According to our meta-analysis, phonosurgery is the first option to be considered in VFP treatment, but conservative voice rehabilitation plays a crucial role too. Its duration and type vary widely and post-surgery VT longer than 3 weeks seems to be more effective than shorter VT (see Tables S6—S9, Supplementary Materials). After phonosurgery, patients are usually prescribed vocal rest. However, standards on its reasonable duration and the type of vocal utterances that can be allowed during this period are lacking [57,58,59]. For optimal vocal outcome, postoperative VT that includes vocal hygiene and a few weeks of VT with vocal function exercises, resonant voice, or other exercise programs described above seems reasonable. A hierarchy of effective voice exercises, starting with a soft voice with little impact on the vocal folds and progressing to a loud voice for robust daily voice use, should be compiled and researched.

## 5. Conclusions

In our meta-analysis, phonosurgery alone and phonosurgery followed by voice therapy are effective in treating dysphonia due to vocal fold polyps. Both phonosurgery alone and phonosurgery with subsequent voice therapy can result in specific voice-related outcomes; thus, the type of therapy can be chosen according to the results of the assessment parameters after phonosurgery. In particular, additional voice therapy should be considered if a hoarse or unstable voice is still present after phonosurgery. If there is uncertainty in the clinical diagnosis about the possible dignity of the polyp mass, phonosurgery should be performed.

Further research on vocal hygiene and rehabilitation strategies after phonosurgery and on treatment effects according to the size and other morphological characteristics of vocal fold polyps is recommended.

In addition, further studies and meta-analyses are needed to account for polyp size, form, mass, length, and impact on glottic configuration in determining whether phonosurgery, voice therapy, or a combination treatment is most helpful.

## Figures and Tables

**Table 1:** Characteristics of clinical trials in the meta-analysis.

Study	Type and Features of VFP	N	Intervention Groups	Interventions with Durations Between Pre- and Post-Treatment	Duration of Voice Therapy in Weeks	Outcome Measures
Kim et al. (1999) [22]	All types and features of VFP	58	Phonosurgery	Treatment method: cold knife Duration: 2 months	n.a.	Jitter Shimmer NHR MPT
Kim & Auo (2008) [23]	All types and features of VFP	62	Phonosurgery	Treatment method: 585 nm pulsed dye laser (office-based) Duration: 2 months	n.a.	Jitter Shimmer NHR G, R, B
Kluch & Olszewski (2008) [24]	All types and features of VFP	16	Combination of phonosurgery and voice therapy	Treatment method: cold knife or CO_2_ laser using microlaryngoscopy under general anesthesia, and breathing and voice exercises Duration: > 1 month	4 weeks	G, R, B
Kim et al. (2008) [25]	Unilateral; all sizes and features of VFP	8	Phonosurgery	Treatment method: cold knife Duration: 2–3 months	n.a.	VHI-T
Kluch & Olszewski (2009) [26]	All types and features of VFP	25	Combination of phonosurgery and voice therapy	Treatment method: cold knife or CO_2_ laser using microlaryngoscopy under general anesthesia, and breathing and voice exercises Duration: >1 month	4 weeks	G, R, B
Kim et al. (2009) [27]	Small VFP	33	Voice therapy	Treatment method: Vocal hygiene and Seong-Tae Kim’s multiple voice therapy technique Duration: >1 month	4 to 16 weeks	Jitter Shimmer NHR MPT
Choi et al. (2011) [28]	All types and features of VFP	128	Phonosurgery	Treatment method: cold knife Duration: >1 month	n.a.	Jitter Shimmer NHR MPT
Petrovic-Lazic et al. (2011) [29]	All types and features of VFP	46	Phonosurgery	Treatment method: cold knife Duration: 3 weeks	n.a.	Jitter Shimmer NHR
Ju et al. (2013) [30]	All types and features of VFP	118	(a) Phonosurgery (n = 63) (b) Combination of phonosurgery and voice therapy (n = 55)	(a) Treatment method: cold knife (a) Duration: 2 months (b) Treatment method: cold knife, vocal hygiene, and resonant voice (b) Duration: 2 months	(a) n.a. (b) 1 week	Jitter Shimmer NHR MPT VHI-T, VHI-P, VHI-F, VHI-E G, R, B
Schindler et al. (2013) [31]	All types and features of gelatinous VFP	20	Voice therapy	Treatment method: vocal hygiene, abdominal breathing, resonant voice, yawn sigh approach, and manual therapy Duration: 1–2 months	1 to 2 months	Jitter Shimmer NHR MPT VHI-T, VHI-P, VHI-F, VHI-E G, R, B
Choe et al. (2013) [32]	Unilateral; no giant VFP but other types and features	41	Combination of phonosurgery and voice therapy (two groups)	Treatment method: CO_2_ laser using microlaryngoscopy under general anesthesia or cold knife, vocal hygiene, and resonant voice Duration: 7 weeks	4 weeks	Jitter Shimmer NHR MPT VHI-T, VHI-P, VHI-F, VHI-E
Wang et al. (2013) [33]	Unilateral; hemorrhagic small to medium vocal polyps	36	Phonosurgery (two groups)	Treatment method: KTP laser (office-based) or KTP plus cold knife using microlaryngoscopy under general anesthesia Duration: 6 weeks	n.a.	Jitter Shimmer NHR MPT
Karasu et al. (2014) [34]	All types and features of VFP	51	Phonosurgery (two groups)	Treatment method: diode laser using microlaryngoscopy under general anesthesia or cold knife Duration: 2 months	n.a.	Jitter Shimmer NHR VHI-T, VHI-P, VHI-F, VHI-E
Lin et al. (2014) [35]	Unilateral; all sizes and features of VFP	60	(a) Phonosurgery (n = 30) (b) Combination of phonosurgery and voice therapy (n = 30)	(a) Treatment method: CO_2_ laser using microlaryngoscopy under general anesthesia (a) Duration: 5–12 weeks (b) Treatment method: CO_2_ laser using microlaryngoscopy under general anesthesia, vocal hygiene, relaxation training, breathing training, yawn sigh approach, chewing approach, and tone sandhi pronunciation (b) Duration: 5–12 weeks	(a) n.a. (b) 4 weeks	Jitter Shimmer NHR MPT VHI-T G, R, B
Wang et al. (2015) [36]	Small to medium sizes and all features of VFP	34	Phonosurgery (two groups)	Treatment method: KTP2 laser (office-based) or cold knife Duration: 6 weeks	n.a.	Jitter Shimmer NHR MPT
Mizuta et al. (2015) [37]	All types and features of VFP	54	Phonosurgery (two groups)	Treatment method: angiolytic laser (office-based) or cold knife Duration: 6 weeks	n.a.	Jitter Shimmer MPT
Petrovic-Lazic et al. (2015) [38]	Medium sizes and all features of VFP	41	Combination of phonosurgery and voice therapy	Treatment method: cold knife and voice therapy Duration: 6 weeks	4 weeks	Jitter Shimmer NHR G, R, B
Zhang et al. (2015) [39]	Bilateral; all sizes and features of VFP	60	Phonosurgery (two groups)	Treatment method: CO_2_ laser using microlaryngoscopy under general anesthesia or cold knife Duration: 3 months	n.a.	Jitter Shimmer NHR MPT VHI-T G, R, B
Lee et al. (2016) [40]	Unilateral; all sizes and diffuse or pedunculated growths of VFP	23	Phonosurgery	Treatment method: cold knife Duration: 2 months	n.a.	Jitter Shimmer NHR MPT VHI-T
Zhuge et al. (2016) [41]	Small fusiform translucent bulge unilateral or bilateral VFP located at the junction of 1/3 of the front and the middle of the vocal fold	66	Voice therapy	Treatment method: relaxation training, breathing exercises, vocal function exercises, resonant voice, and vocal hygiene Duration: 3 months	12 weeks	MPT VHI-T, VHI-P, VHI-F, VHI-E
Barillari et al. (2017) [42]	Unilateral; all sizes and features of the VFP at the free edge of the vocal fold	140	(a) Voice therapy (n = 70) (b) Combination of phonosurgery and voice therapy (n = 70)	(a) Treatment method: voice therapy expulsion (a) Duration: 3 months (b) Treatment method: CO_2_ laser using microlaryngoscopy under general anesthesia, vocal hygiene, relaxation training, vocal function exercises, and breath support (b) Duration: 6 weeks	(a) 12 weeks (b) 4 weeks	VHI-T G
You et al. (2017) [43]	VFP that were smooth and had translucent pedunculated neoplasm or fusiform translucent smooth neoplasm with a wider base by the free edge of the vocal folds	96	(a) Phonosurgery (n = 41) (b) Combination of phonosurgery and voice therapy (n = 55)	(a) Treatment method: cold knife (a) Duration: 4 months (b) Treatment method: cold knife, vocal hygiene, relaxation training, vocal function exercises, breathing exercises, and resonant voice (b) Duration: 4 months	(a) n.a. (b) 12 weeks	MPT VHI-T, VHI-P, VHI-F, VHI-E
Lin et al. (2018) [44]	All types and features of VFP	90	Phonosurgery (two groups)	Treatment method: KTP laser (office-based) or cold knife Duration: 1–2 months	n.a.	Jitter Shimmer NHR MPT
Sahin et al. (2018) [45]	All types and features of VFP	165	(a) Phonsurgery (n = 138) (b) Voice therapy (n = 27)	(a) Treatment method: cold knife (a) Duration: > 3 months (b) Treatment method: vocal hygiene, relaxation training, vocal function exercises, breathing and posture exercises, and resonant voice (b) Duration: 4 months	(a) n.a. (b) 16 weeks	Jitter Shimmer NHR G, R, B
Oh et al. (2018) [46]	Unilateral; all sizes and features of VFP	130	(a) Phonsurgery (n= 44) (b) Combination of phonosurgery and voice therapy (two groups; n = 86)	(a) Treatment method: cold knife (a) Duration: 1 month (b) Treatment method: cold knife and vocal hygiene or cold knife and relaxation training, vocal function exercises, breathing exercises, resonant voice, and manual therapy (b) Duration: 4 months	(a) n.a. (b) 1–4 weeks	Jitter Shimmer NHR MPT VHI-T, VHI-P, VHI-F, VHI-E G, R, B,
Wang et al. (2019) [47]	Small fusiform translucent bulge VFP’s located at the junction of 1/3 of the front and the middle of the vocal fold	69	(a) Phonsurgery (n = 31) (b) Voice therapy (n = 38)	(a) Treatment method: cold knife (a) Duration: 4 months (b) Treatment method: vocal hygiene, relaxation training, breathing and posture training, and vocal acoustic training (b) Duration: 4 months	(a) n.a. (b) 12 weeks	MPT VHI-T, VHI-P, VHI-F, VHI-E
Prasad et al. (2020) [48]	All types and features of VFP	40	Phonosurgery	Treatment method: cold knife Duration: 3 months	n.a.	MPT
Kim et al. (2020) [49]	Unilateral; all sizes and features of VFP	20	Phonosurgery	Treatment method: cold knife Duration: 1 month	n.a.	Jitter Shimmer NHR
Ma et al. (2021) [50]	All types and features of VFP	25	Phonosurgery	Treatment method: KTP laser (office-based) Duration: 3–14 months	n.a.	Jitter Shimmer MPT VHI-T, VHI-P, VHI-F, VHI-E
Lee et al. (In Press) [51]	All types and features of VFP	72	Phonosurgery	Treatment method: cold knife Duration: 10–14 days	n.a.	VHI-T, VHI-P, VHI-F, VHI-E
Kang et al. (In Press) [52]	Unilateral; all sizes and features of VFP	77	Phonosurgery	Treatment method: cold knife Duration: 6 weeks	n.a.	Jitter Shimmer NHR MPT VHI-T, VHI-P, VHI-F, VHI-E G, R, B

VFP: Vocal fold polyp; G: grade/hoarseness; R: roughness; B: breathiness; NHR: noise-to-harmonics ratio; MPT: maximum phonation time; VHI: the 30-item Voice Handicap Index (VHI—30) which outputs three subscales with statements on physical (P), functional (F), and emotional (E) domains and a total score (T).

## Data Availability

The original contributions presented in the study are included in the article; further inquiries can be directed to the corresponding author.

## Supplementary Materials

The following supporting information can be downloaded at https://www.mdpi.com/article/10.3390/jcm12103451/s1, Table S1: Systematic search strategies; Table S2: Risk of bias analysis of RCT and observational studies; Table S3: Meta-analyses by treatment and by voice measures (random effects model), with heterogeneity index I² and Egger’s publication bias test; Table S4: Meta-analysis for Cohen’s *d* (random effects model); Table S5: Network meta-analysis; Table S6: Subgroup meta-analysis for perceived voice quality level; Table S7: Subgroup meta-analysis for acoustics; Table S8: Subgroup meta-analysis for MPT; Table S9: Subgroup meta-analysis for VHI-30; Table S10: Influence of morphological polyp characteristics on the MPT outcome of both phonosurgery and VT.
