# Supplementary material for: The Efficacy of Different Voice Treatments for Vocal Fold Polyps: A Systematic Review and Meta-Analysis
Source: J Clin Med. 2023 May 13;12(10):3451. doi: 10.3390/jcm12103451 (PMC10219546; doi:10.3390/jcm12103451)
Supplement: Supplementary file 1 [file jcm-12-03451-s001.zip › jcm-2380888-supplementary.pdf]

SUPLLEMENTAL MATERIAL

Table S1. Systematic search strategies

Medline

("vocal fold polyp"[All Fields] OR "vocal polyp"[All Fields] OR ("polyp s"[All Fields] OR "polypous"[All Fields] OR "polyps"[MeSH Terms] OR "polyps"[All Fields] OR "polyp"[All Fields])) AND ("voice disorder"[All Fields] OR ("dysphonia"[MeSH Terms] OR "dysphonia"[All Fields] OR "dysphonias"[All Fields]) OR "benign vocal fold pathology"[All Fields] OR "organic voice disorder"[All Fields]) AND ("voice therapy"[All Fields] OR "voice treatment"[All Fields] OR ("laser s"[All Fields] OR "lasers"[MeSH Terms] OR "lasers"[All Fields] OR "laser"[All Fields] OR "lasered"[All Fields] OR "lasering"[All Fields]) OR "phonosurgery"[All Fields] OR ("surgery"[MeSH Subheading] OR "surgery"[All Fields] OR "surgical procedures, operative"[MeSH Terms] OR ("surgical"[All Fields] AND "procedures"[All Fields] AND "operative"[All Fields]) OR "operative surgical procedures"[All Fields] OR "general surgery"[MeSH Terms] OR ("general"[All Fields] AND "surgery"[All Fields]) OR "general surgery"[All Fields] OR "surgery s"[All Fields] OR "surgerys"[All Fields] OR "surgeries"[All Fields]) OR ("microflap"[All Fields] OR "microflaps"[All Fields]) OR "cold knife"[All Fields] OR "voice training"[All Fields] OR "vocal treatment"[All Fields])

CENTRAL

#1 ("vocal fold polyp"):ti,ab,kw OR ("vocal polyp"):ti,ab,kw OR ("polyp"):ti,ab,kw
#2 ("voice therapy"):ti,ab,kw OR ("voice treatment"):ti,ab,kw OR ("voice training"):ti,ab,kw OR ("vocal treamtent"):ti,ab,kw
#3 ("dysphonia"):ti,ab,kw OR ("voice disorder"):ti,ab,kw OR ("benign vocal fold pathology"):ti,ab,kw OR ("organic voice disorder"):ti,ab,kw
#4 ("phonosurgery"):ti,ab,kw OR ("surgery"):ti,ab,kw OR ("laser"):ti,ab,kw OR ("cold knife"):ti,ab,kw OR ("microflap"):ti,ab,kw
#5 #1 AND #2
#6 #1 AND #3 AND #4

KoreaScience

#1 “vocal polyp” AND therapy
#2 “vocal polyp” AND surgery

CINAHL

S1 "vocal fold polyp" OR "Vocal polyp" OR polyp
S2 "Voice therapy" OR "vice treatment" OR "voice training" OR "vocal treatment"
S3 "dysphonia" OR "voice disorder" OR "benign vocal fold pathology" OR "organic voice disorder"
S4 "phonosurgery" OR "surgery" OR "laser" OR "cold knife"
S5 (S1 AND S2)
S6 (S1 AND s3 AND S4)

Table S2. Risk of bias analysis of RCT and observational studies [22-52]

Risk of bias domains

Study	D1	D2	D3	D4	D5	Overall
Lin et al. (2014)	+	-	+	+	-	-
Zhang et al. (2015)	-	+	+	+	-	-
Barillari et al. (2017)	+	-	+	+	+	+

Domains:
D1: Bias arising from the randomization process.
D2: Bias due to deviations from intended intervention.
D3: Bias due to missing outcome data.
D4: Bias in measurement of the outcome.
D5: Bias in selection of the reported result.

Judgement
- Some concerns
+ Low

Risk of bias domains

Study	D1	D2	D3	D4	D5	D6	D7	Overall
Kim et al. (1999)	X	-	-	+	X	+	X	X
Kim & Auo (2008)	X	X	-	+	-	-	X	X
Kluch & Olszewski (2008)	X	X	X	+	+	X	X	X
Kim et al. (2008)	X	X	-	+	+	X	X	X
Kluch & Olszewski (2009)	X	X	X	+	+	X	X	X
Kim et al. (2009)	+	-	-	+	X	+	X	X
Choi et al. (2011)	-	-	-	+	+	+	-	-
Petrovic-Lazic et al. (2011)	+	+	-	+	+	+	+	-
Ju et al. (2013)	-	X	+	+	+	-	+	X
Schindler et al. (2013)	X	-	+	+	+	+	-	X
Choe et al. (2013)	-	X	-	+	X	-	+	X
Wang et al. (2013)	-	+	-	+	+	+	+	-
Karasu et al. (2014)	X	+	-	+	+	-	-	X
Wang et al. (2015)	-	X	-	+	+	+	-	X
Mizuta et al. (2015)	-	+	+	+	+	+	-	-
Petrovic-Lazic et al. (2015)	-	+	X	+	+	-	-	X
Lee et al. (2016)	+	+	+	+	+	+	+	+
Zhuge et al. (2016)	+	+	+	+	-	-	+	-
You et al. (2017)	+	+	+	+	+	-	+	-
Lin et al. (2018)	-	X	+	+	X	-	-	X
Sahin et al. (2018)	-	X	+	+	X	+	-	X
Oh et al. (2018)	-	-	+	+	X	-	-	X
Wang et al. (2019)	-	X	+	+	+	-	-	X
Prasad et al. (2020)	X	-	+	+	+	+	-	X
Kim et al. (2020)	X	-	-	+	+	+	-	X
Ma et al. (2021)	+	+	-	+	+	-	-	-
Lee et al. (In Press)	-	+	-	+	-	-	+	-
Kang et al. (In Press)	+	+	+	+	+	-	+	-

Domains:
D1: Bias due to confounding.
D2: Bias due to selection of participants.
D3: Bias in classification of interventions.
D4: Bias due to deviations from intended interventions.
D5: Bias due to missing data.
D6: Bias in measurement of outcomes.
D7: Bias in selection of the reported result.

Judgement
X Serious
- Moderate
+ Low

Table S3. Meta-analysis by treatment and by voice measures (random effects model) with heterogeneity index I^2 and Egger's publication bias test [22-52]

Voice parameters	Treatment approaches (n)	Parameter	Mean difference from pre- to post-treatment (95% CI)	*P* value	I^2	*P* value Egger's test
Auditory-perceptual judgment	Phonosurgery (n=474)	G	-1.256 (-1.569 to -.944)	**<.001**	97.83%	.333
	Voice Therapy (n=117)		-1.223 (-2.293 to -.152)	**.025**	99.44%	.939
	Combined Treatment (n=350)		-1.504 (-1.972 to -1.037)	**<.001**	98.57%	.372
	Phonosurgery (n=474)	R	-1.189 (-1.505 to -0.872)	**<.001**	98.08%	.439
	Voice Therapy (n=47)		-.552 (-1.198 to .093)	.093	92.12%	**<.001**
	Combined Treatment (n=280)		-1.041 (-1.361 to -.722)	**<.001**	95.58%	.551
	Phonosurgery (n=474)	B	-1.080 (-1.529 to -.630)	**<.001**	99.35%	.098
	Voice Therapy (n=47)		-.220 (-0.327 to -.113)	**<.001**	0.00%	**<.001**
	Combined Therapy (280)		-1.055 (-1.557 to -.553)	**<.001**	98.40%	**.018**
Acoustics	Phonosurgery (n=1039)	Jitter	-1.266% (-1.663 % to -.869 %)	**<.001**	98.18%	**.045**
	Voice Therapy (n=80)		-.494 % (-.932 % to -.565 %)	**<.001**	83.20%	.293
	Combined Treatment (n=253)		-1.457 % (-1.615 % to -1.299 %)	**<.001**	0.00%	.722
	Phonosurgery (n=1039)	Shimmer	-2.300 % (-3.061 % to -1.539 %)	**<.001**	97.77%	.171
	Voice Therapy (n=80)		-1.487 % (-3.065 % to .915 %)	**<.001**	93.26%	**.026**
	Combined Treatment (253)		-3.181 % (-3.950 % to -2.413 %)	**<.001**	84.12%	.758
	Phonosurgery (n=960)	NHR	-.087 dB (-.113 dB to -.061 dB)	**<.001**	97.54%	.059
	Voice Therapy (n=80)		-.068 dB (-.118 dB to -.017 dB)	**<.001**	92.73%	.111
	Combined Treatment (253)		-.077 dB (-.096 dB to -.059 dB)	**<.001**	60.36%	.345
Aerodynamics	Phonosurgery (n=834)	MPT	3.265 sec (2.203 sec to 4.328 sec)	**<.001**	92.44%	.893
	Voice Therapy (n=157)		2.561 sec (1.355 sec to 3.766 sec)	**<.001**	65.80%	.892
	Combined Treatment (n=267)		4.065 sec (2.045 sec to 6.084 sec)	**<.001**	93.52%	.384
VHI-30	Phonosurgery (n=404)	E	-7.072 (-10.786 to -3.357)	**<.001**	96.54%	.337
	Voice Therapy (n=124)		-3.093 (-4.440 to -1.747)	**<.001**	0.00%	.094
	Combined Treatment (n=237)		-6.242 (-11.913 to -0.571)	**.031**	94.04%	**<.001**
	Phonosurgery (n=404)	F	-7.437 (-11.389 to -3.485)	**<.001**	97.49%	.146
	Voice Therapy (n=124)		-2.731 (-4.162 to -1.300)	**<.001**	48.39%	.969
	Combined Treatment (n=237)		-5.239 (-7.124 to -3.354)	**<.001**	87.68%	.552
	Phonosurgery (n=404)	P	-10.463 (-15.829 to -5.096)	**<.001**	98.13%	.146
	Voice Therapy (n=124)		-5.022 (-6.569 to -3.476)	**<.001**	0%	**.027**
	Combined Treatment (n=237)		-12.200 (-16.668 to -7.731)	**<.001**	95.97%	.369
	Phonosurgery (n=525)	T	-22.753 (-29.266 to -16.240)	**<.001**	96.99%	.256
	Voice Therapy (n=194)		-18.886 (-42.996 to 5.224)	.125	99.12%	**.017**
	Combined Treatment (n=337)		-22.896 (-33.529 to -12.264)	**<.001**	99.46%	**<.001**

Bold marked p-values indicate significance (p< 0.05)

Table S4. Meta-analysis for Cohen's *d* (random effecs model) [22-52]

Assessment categories	Treatment approaches	Parameter	Cohen's *d* (95% CI)	*P* value
Auditory-perceptual judgment	Phonosurgery (n=474)	G	19.366 (9.825 to 28.907)	**<.001**
	Voice Therapy (n=117)		18.327 (1.100 to 35.553)	**.037**
	Combined Treatment (n=350)		13.109 (8.895 to 17.323)	**<.001**
	Phonosurgery (n=474)	R	20.551 (-2.520 to 43.622)	.081
	Voice Therapy (n=47)		9.497 (-6.924 to 25.919)	.257
	Combined Treatment (n=280)		10.548 (6.478 to 14.617)	**<.001**
	Phonosurgery (n=474)	B	19.454 (12.625 to 26.282)	**<.001**
	Voice Therapy (n=47)		2.720 (0.908 to 4.531)	**.003**
	Combined Therapy (280)		9.995 (5.515 to 14.475)	**<.001**
Acoustics	Phonosurgery (n=1039)	Jitter	7.602 (4.581 to 10.623)	**<.001**
	Voice Therapy (n=80)		3.742 (1.884 to 5.599)	**<.001**
	Combined Treatment (n=253)		6.399 (3.625 to 9.174)	**<.001**
	Phonosurgery (n=1039)	Shimmer	6.744 (3.813 to 9.675)	**<.001**
	Voice Therapy (n=80)		3.475 (0.807 to 6.144)	**<.001**
	Combined Treatment (253)		7.810 (4.132 to 11.489)	**<.001**
	Phonosurgery (n=960)	NHR	5.975 (3.857 to 8.093)	**<.001**
	Voice Therapy (n=80)		4.017 (2.930 to 5.104)	**<.001**
	Combined Treatment (253)		4.716 (2.527 to 6.905)	**<.001**
Aerodynamics	Phonosurgery (n=834)	MPT	4.627 (2.014 to 7.240)	**.001**
	Voice Therapy (n=157)		3.807 (1.954 to 5.660)	**<.001**
	Combined Treatment (n=267)		4.912 (0.628 to 9.196)	**.025**
VHI-30	Phonosurgery (n=404)	E	6.285 (3.132 to 9.437)	**<.001**
	Voice Therapy (n=124)		2.780 (1.433 to 4.127)	**<.001**
	Combined Treatment (n=237)		5.817 (4.970 to 6.663)	**<.001**
	Phonosurgery (n=404)	F	6.829 (3.629 to 10.030)	**<.001**
	Voice Therapy (n=124)		3.022 (1.376 to 4.669)	**<.001**
	Combined Treatment (n=237)		6.310 (4.176 to 8.444)	**<.001**
	Phonosurgery (n=404)	P	9.037 (4.451 to 13.622)	**<.001**
	Voice Therapy (n=124)		3.944 (1.898 to 5.991)	**<.001**
	Combined Treatment (n=237)		10.605 (7.201 to 14.008)	**<.001**
	Phonosurgery (n=525)	T	9.737 (6.826 to 12.647)	**<.001**
	Voice Therapy (n=194)		60.150 (-97.424 to 217.724)	.454
	Combined Treatment (n=337)		35.737 (-46.940 to 118.415)	.397

Bold marked p-values indicate significance (p< 0.05)

Table S5. Network meta-analysis [22-52]

Assessment categories	Parameter	Treatment approaches	Comparison	*P* value
Auditory-perceptual judgement	G	Phonosurgery (n=474)	Phonosurgery vs. Voice Therapy	.518
		Voice Therapy (n=117)	Phonosurgery vs. Combined Treatment	**<.001**
		Combined Treatment (n=350)	Voice Therapy vs. Combined Treatment	**<.001**
	R	Phonosurgery (n=474)	Phonosurgery vs. Voice Therapy	**<.001**
		Voice Therapy (n=47)	Phonosurgery vs. Combined Treatment	**<.001**
		Combined Treatment (n=280)	Voice Therapy vs. Combined Treatment	**<.001**
	B	Phonosurgery (n=474)	Phonosurgery vs. Voice Therapy	**<.001**
		Voice Therapy (n=47)	Phonosurgery vs. Combined Treatment	.198
		Combined Therapy (280)	Voice Therapy vs. Combined Treatment	**<.001**
Acoustics	Jitter	Phonosurgery (n=1039)	Phonosurgery vs. Voice Therapy	**<.001**
		Voice Therapy (n=80)	Phonosurgery vs. Combined Treatment	**<.001**
		Combined Treatment (n=253)	Voice Therapy vs. Combined Treatment	**<.001**
	Shimmer	Phonosurgery (n=1039)	Phonosurgery vs. Voice Therapy	**<.001**
		Voice Therapy (n=80)	Phonosurgery vs. Combined Treatment	**<.001**
		Combined Treatment (253)	Voice Therapy vs. Combined Treatment	**<.001**
	NHR	Phonosurgery (n=960)	Phonosurgery vs. Voice Therapy	**<.001**
		Voice Therapy (n=80)	Phonosurgery vs. Combined Treatment	**<.001**
		Combined Treatment (253)	Voice Therapy vs. Combined Treatment	**<.001**
Aerodynamics	MPT	Phonosurgery (n=834)	Phonosurgery vs. Voice Therapy	**<.001**
		Voice Therapy (n=157)	Phonosurgery vs. Combined Treatment	**<.001**
		Combined Treatment (n=267)	Voice Therapy vs. Combined Treatment	**<.001**
VHI-30	E	Phonosurgery (n=404)	Phonosurgery vs. Voice Therapy	**<.001**
		Voice Therapy (n=124)	Phonosurgery vs. Combined Treatment	**<.001**
		Combined Treatment (n=237)	Voice Therapy vs. Combined Treatment	**<.001**
	F	Phonosurgery (n=404)	Phonosurgery vs. Voice Therapy	**<.001**
		Voice Therapy (n=124)	Phonosurgery vs. Combined Treatment	**<.001**
		Combined Treatment (n=237)	Voice Therapy vs. Combined Treatment	**<.001**
	P	Phonosurgery (n=404)	Phonosurgery vs. Voice Therapy	**<.001**
		Voice Therapy (n=124)	Phonosurgery vs. Combined Treatment	**<.001**
		Combined Treatment (n=237)	Voice Therapy vs. Combined Treatment	**<.001**
	T	Phonosurgery (n=525)	Phonosurgery vs. Voice Therapy	**<.001**
		Voice Therapy (n=194)	Phonosurgery vs. Combined Treatment	.674
		Combined Treatment (n=337)	Voice Therapy vs. Combined Treatment	**<.001**

Bold marked p-values indicate significance (p< 0.05)

Table S6. Subgroup meta-analysis for perceived voice quality level [23,24,26,30,35,38,39,42,45,46,52]

Parameter	Treatment approaches	Subgroup	Subgroup definition	Mean difference from pre- to post-treatment	*P* value	I^2	*P* value Egger's test
G	Phonosurgery (n = 474)	time period between the pre and post measurement	≤ 1 month	-.895 (-2.473 to 0.683)	.266	99.42%	**<.001**
			1 to 2 months	-1.358 (-1.782 to -0.933)	**<.001**	96.88%	.665
			≥ 3 months	-1.400 (-1.607 to -1.193)	**<.001**	87.05%	.160
		type of surgical technique	cold knife	-1.378 (-1.624 to -1.132)	**<.001**	95.12%	.578
			Laser	-1.051 (-1.974 to -.127)	**.026**	99.04%	.525
			combination of cold knife and laser	n.a	n.a	n.a	n.a
	Combined Treatment (n = 350)	duration of voice therapy	1 to 2weeks	-1.638 (-1.810 to -1.466)	**<.001**	64.96%	.303
			longer than 3 weeks	-1.383 (-2.180 to -.587)	**.001**	99.24%	.651
		type of surgical technique	cold knife	-1.593 (-2.053 to -1.133)	**<.001**	98.03%	**.014**
			laser	-1.183 (-3.162 to .797)	.242	99.52%	**<.001**
R	Phonosurgery (n = 474)	time period between the pre and post measurement	≤ 1 month	-.914 (-2.512 to -.683)	.262	99.44%	**<.001**
			1 to 2 months	-1.263 (-1.816 to -0.710)	**<.001**	97.76%	.777
			≥ 3 months	-1.329 (-1.437 to -1.221)	**<.001**	54.11%	.153
		type of surgical technique	cold knife	-1.264 (-1.516 to -1.012)	**<.001**	95.13%	.529
			laser	-1.060 (-2.054 to -0.0663)	**.037**	99.22%	.709
			combination of cold knife and laser	n.a	n.a	n.a	n.a
	Combined Treatment (n = 280)	duration of voice therapy	1 to 2weeks	-.821 (-1.315 to -.328)	**.001**	97.50%	.084
			longer than 3 weeks	-.917 (-1.453 to -.380)	**.001**	98.13%	.559
		type of surgical technique	cold knife	-1.019 (-1.390 to -.648)	**<.001**	97.58%	.142
			laser	n.a	n.a	n.a	n.a
B	Phonosurgery (n = 474)	time period between the pre and post measurement	≤ 1 month	-.936 (-1.808 to -.063)	**.036**	96.80%	**<.001**
			1 to 2 months	-.958 (-1.194 to -.723)	**<.001**	88.07%	.338
			≥ 3 months	-1.296 (-2.240 to -.351)	**.007**	99.80%	.094
		type of surgical technique	cold knife	-1.125 (-1.707 to -.542)	**<.001**	99.46%	.153
			laser	-1.004 (-1.755 to -.254)	**.009**	98.61%	.259
			combination of cold knife and laser	n.a	n.a	n.a	n.a
	Combined Treatment (n = 280)	duration of voice therapy	1 to 2weeks	-.887 (-1.570 to -.205)	**.011**	98.25%	.278
			longer than 3 weeks	-1.225 (-2.129 to -.321)	**.008**	98.87%	.127
		type of surgical technique	cold knife	-1.029 (-1.578 to -.480)	**<.001**	98.59%	**.019**
			laser	n.a	n.a	n.a	n.a

Bold marked p-values indicate significance (p< 0.05)

Table S7. Subgroup meta-analysis for acoustics [22,23,28-30,32-40,44-46,49,50,52]

Parameter	Treatment approaches	Subgroup	Subgroup definition	Mean difference from pre- to post-treatment	*P* value	I^2	*P* value Egger's test
Jitter	Phonosurgery (n = 1039)	time period between the pre and post measurement	≤ 1 month	-1.027 % (-1.593 % to -.461 %)	**<.001**	94.07%	.452
			1 to 2 months	-1.066 % (-1.410 % to -.721 %)	**<.001**	92.62%	**.012**
			≥ 3 months	-2.166 % (-3.925 % to -.408 %)	**.016**	99.69%	.187
		type of surgical technique	cold knife	-1.490 % (-2.101 % to -.879 %)	**<.001**	98.02%	.324
			laser	-1.267 % (-2.274 % to -.259 %)	**.014**	98.51%	.958
			combi of cold knife and laser	-.686 % (-0.945 % to -.426 %)	**<.001**	.00%	.456
	Combined Treatment (n = 253)	duration of voice therapy	1 to 2weeks	-1.637 % (-2.017 % to -1.256 %)	**<.001**	.00%	**<.001**
			longer than 3 weeks	-1.419 % (-1.593 % to -1.246 %)	**<.001**	.00%	.901
		type of surgical technique	cold knife	-1.521 % (-1.702 % to -1.341 %)	**<.001**	.00%	.422
			Laser	-1.247 % (-1.573 % to -.922 %)	**<.001**	.00%	**<.001**
Shimmer	Phonosurgery (n = 1039)	time period between the pre and post measurement	≤ 1 month	-2.930 % (-6.652 % to 0.793 %)	.123	99.42%	.529
			1 to 2 months	-2.195 % (-3.043 % to -1.347 %)	**<.001**	94.78%	.133
			≥ 3 months	-2.646 % (-5.039 % to -.252 %)	**.030**	99.33%	.209
		type of surgical technique	cold knife	-2.390 % (-3.434 % to -1.346 %)	**.001**	97.91%	.149
			laser	-2.700 % (-4.990 % to -0.555 %)	**.014**	98.54%	.957
			combi of cold knife and laser	-0.777 % (-1.394 % to -0.160 %)	**.014**	54.21%	.079
	Combined Treatment (n = 253)	duration of voice therapy	1 to 2weeks	-2.500 % (-4.705 % to -0.372 %)	**.022**	80.35%	**<.001**
			longer than 3 weeks	-3.415 % (-4.256 % to -2.574 %)	**<.001**	85.41%	.535
		type of surgical technique	cold knife	-3.459 % (-4.442 % to -2.477 %)	**<.001**	80.92%	.891
			laser	-2.428 % (-2.844 % to -2.013 %)	**<.001**	.00%	**<.001**
NHR	Phonosurgery (n = 960)	time period between the pre and post measurement	≤ 1 month	-.040 dB (-.049 dB to -.031 dB)	**<.001**	40.22%	.626
			1 to 2 months	-.043 dB (-.058 dB to -.027 dB)	**<.001**	83.80%	.119
			≥ 3 months	-.339 dB (-.452 dB to -.225 dB)	**<.001**	96.98%	.231
		type of surgical technique	cold knife	-.105 dB (-.137 dB to -.073 dB)	**<.001**	97.64%	.058
			laser	-.079 dB (-.157 dB to -.002 dB)	**.045**	98.52%	.555
			combi of cold knife and laser	-.035 dB (-.050 dB to -.020 dB)	**<.001**	0.00%	.654
	Combined Treatment (n = 253)	duration of voice therapy	1 to 2weeks	-.067 dB (-.092 dB to -.042 dB)	**<.001**	25.08%	**<.001**
			longer than 3 weeks	-.080 dB (-.104 dB to -.056 dB)	**<.001**	62.95%	.150
		type of surgical technique	cold knife	-.075 dB (-.092 dB to -.058 dB)	**<.001**	55.20%	.761
			Laser	.093 dB (-.368 dB to .555 dB)	.692	80.27%	**<.001**

Bold marked p-values indicate significance (p< 0.05)

Table S8. Subgroup meta-analysis for MPT [22,28,30,32,33,35-37,39,40,43,44,46-48,50,52]

Treatment approaches	Subgroup	Subgroup definition	Mean difference from pre- to post-treatment	*P* value	I^2	*P* value Egger's test
Phonosurgery (n = 834)	time period between the pre and post measurement	≤ 1 month	2.244 sec (-0.361 sec to 4.849 sec)	.091	95.65%	.287
		1 to 2 months	3.249 sec (2.405 sec to 4.093 sec)	**<.001**	52.90%	.943
		≥ 3 months	4.005 sec (1.955 sec to 6.055 sec)	**<.001**	95.58%	.468
	type of surgical technique	cold knife	3.304 sec (1.992 sec to 4.615 sec)	**<.001**	91.51%	.363
		laser	2.788 sec (0.098 sec to 5.478 sec)	**.042**	95.61%	.780
		combination of cold knife and laser	3.879 sec (2.930 sec to 4.827 sec)	**<.001**	.00%	.069
Combined Treatment (n = 267)	duration of voice therapy	1 to 2weeks	3.241 sec (1.722 sec to 4.760 sec)	**<.001**	63.29%	**<.001**
		longer than 3 weeks	4.521 sec (1.436 sec to 7.606 sec)	**.004**	95.55%	.537
	type of surgical technique	cold knife	3.786 sec (1.089 sec to 6.482 sec)	**.006**	93.74%	.072
		laser	4.468 sec (3.632 sec to 5.303 sec)	**<.001**	7.26%	**<.001**

Bold marked p-values indicate significance (p< 0.05)

Table S9. Subgroup meta-analysis for VHI-30 [25,30,32,34,35,39,40,42,43,46,47,50-52]

Parameter	Treatment approaches	Subgroup	Subgroup definition	Mean difference from pre- to post-treatment (95% CI)	*P* value	I^2	*P* value Egger's test
E	Phonosurgery (n = 404)	time period between the pre and post measurement	≤ 1 month	-3.114 (-6.305 to 0.0778)	.056	88.86%	.497
			1 to 2 months	-11.106 (-17.278 to -4.935)	**<.001**	97.47%	**.042**
			≥ 3 months	n.a	n.a	n.a	n.a
		type of surgical technique	cold knife	-5.991 (-9.871 to -2.111)	**.002**	96.36%	.446
			laser	-10.956 (-19.225 to -2.687)	**<.001**	93.76%	**<.001**
			combination of cold knife and laser	-11.007 (-19.733 to -2.280)	**.013**	98.27%	.078
	Combined Treatment (n = 237)	duration of voice therapy	1 to 2weeks	-8.133 (-10.220 to -6.047)	**<.001**	35.20%	**<.001**
			longer than 3 weeks	-5.019 (-6.438 to -3.600)	**<.001**	59.56%	.295
		type of surgical technique	cold knife	-6.214 (-8.136 to -4.292)	**<.001**	80.71%	.810
			laser	n.a	n.a	n.a	n.a
F	Phonosurgery (n = 404)	time period between the pre and post measurement	≤ 1 month	-3.697 (-6.835 to -0.560)	**.021**	92.09%	.613
			1 to 2 months	-11.875 (-19.680 to -4.070)	**.003**	98.54%	.064
			≥ 3 months	n.a	n.a	n.a	n.a
		type of surgical technique	cold knife	-6.338 (-10.100 to -2.577)	**.001**	96.88%	.096
			laser	n.a	n.a	n.a	n.a
			combination of cold knife and laser	n.a	n.a	n.a	n.a
	Combined Treatment (n = 237)	duration of voice therapy	1 to 2weeks	-6.708 (-7.882 to -5.534)	**<.001**	0.00%	**<.001**
			longer than 3 weeks	-4.576 (-7.064 to -2.088)	**<.001**	89.50%	.659
		type of surgical technique	cold knife	-5.583 (-7.713 to -3.453)	**<.001**	89.53%	.319
			laser	n.a	n.a	n.a	n.a
P	Phonosurgery (n = 404)	time period between the pre and post measurement	≤ 1 month	-3.903 (-5.576 to -2.230)	**<.001**	60.54%	.915
			1 to 2 months	-17.370 (-24.860 to -9.879)	**<.001**	97.97%	.362
			≥ 3 months	n.a	n.a	n.a	n.a
		type of surgical technique	cold knife	-9.138 (-14.849 to -3.426)	**.002**	98.12%	.601
			laser	-15.195 (-27.569 to -2.820)	**.016**	96.28%	**<.001**
			combination of cold knife and laser	n.a	n.a	n.a	n.a
	Combined Treatment (n = 237)	duration of voice therapy	1 to 2weeks	-15.547 (-24.298 to -6.796)	**<.001**	95.86%	**<.001**
			longer than 3 weeks	-10.514 (-15.100 to -5.927)	**<.001**	94.65%	.533
		type of surgical technique	cold knife	-12.378 (-17.472 to -7.285)	**<.001**	96.77%	.273
			laser	n.a	n.a	n.a	n.a
T	Phonosurgery (n = 525)	time period between the pre and post measurement	≤ 1 month	-10.448 (-17.423 to -3.472)	**.003**	92.28%	.272
			1 to 2 months	-35.674 (-52.365 to -18.982)	**<.001**	98.54%	**.007**
			≥ 3 months	-22.682 (-24.917 to -20.448)	**<.001**	0.00%	.686
		type of surgical technique	cold knife	-21.795 (-28,501 to -15,089)	**<.001**	96.47%	.334
			laser	-25.170 (-45.878 to -4.468)	**.017**	98.26%	.747
			combination of cold knife and laser	n.a	n.a	n.a	n.a
	Combined Treatment (n = 337)	duration of voice therapy	1 to 2weeks	-22.527 (-32.364 to -12.689)	**<.001**	95.39%	**<.001**
			longer than 3 weeks	-23.036 (-35.856 to -10.216)	**<.001**	99.48%	**.007**
		type of surgical technique	cold knife	-21.431 (-28.137 to -14.725)	**<.001**	96.31%	**.038**
			laser	-25.457 (-50.368 to -0.546)	**.045**	99.50%	.323

Bold marked p-values indicate significance (p< 0.05)

Table S10. Influence of morphological polyp characteristics on the MPT outcome of both phonosurgery and VT

Study	Therapy	Kind of polyp (size and side)	N	Pre		Post		Mean of the differences from pre to post	
				Mean	SD	Mean	SD	Mean	SD
Wang et al. (2019) [47]	Phonosurgery	Small, 1/3 anteromedian	31	17.28 sec	3.52 sec	17.78 sec	3.99 sec	2.90 sec	1.76 sec
Wang et al. (2015) [36]	Phonosurgery – cold knife	Small to medium	17	10.40 sec	5.40 sec	12.00 sec	4.90 sec		
Wang et al. (2015) [36]	Phonosurgery – laser & cold knife	Small to medium	17	10.30 sec	4.40 sec	14.80 sec	4.80 sec		
Wang et al. (2013) [33]	Phonosurgery– laser	Unilateral hemorrhagic small	16	10.20 sec	5.10 sec	13.90 sec	5.20 sec		
Wang et al. (2013) [33]	Phonosurgery – laser & cold knife	Unilateral hemorrhagic small	20	10.70 sec	4.00 sec	14.90 sec	4.30 sec		
Wang et al. (2019) [2019]	VT	Small, 1/3 anteromedian	38	16.62 sec	3.47 sec	20.09 sec	3.58 sec	2.89 sec	1.11 sec
Zhuge et al. (2016) [41]	VT	Small, 1/3 anteromedian	66	17.13 sec	3.92 sec	18.74 sec	4.09 sec		
Kim et al. (2009) [27]	VT	Small	33	12.60 sec	5.30 sec	16.20 sec	4.60 sec		
